# Increasing and maintaining rates of standardized depression screening in youth with childhood-onset systemic lupus erythematosus in a pediatric rheumatology clinic

**DOI:** 10.1186/s12969-024-01038-3

**Published:** 2025-01-04

**Authors:** Emily Datyner, Jodi Dingle, Victoria Newsome, Lisa H. Buckley, Natasha Belsky, Seungweon Park, Manda Mitchell, Brooke Fine, Barron Patterson, T. Brent Graham, Alaina Davis

**Affiliations:** 1 Department of Pediatrics, Vanderbilt University Medical Center, Monroe Carell Junior Children’s Hospital at Vanderbilt, 2141 Blakemore Avenue, Nashville, TN 37232 USA; 2 Department of Pediatrics, Prisma Health, Columbia, SC USA; 3 North Augusta Pediatrics, North Augusta, SC USA; 4 Vanderbilt University School of Medicine, Nashville, TN USA

**Keywords:** Quality improvement, Childhood onset systemic lupus erythematosus, Depression, Screening

## Abstract

**Background:**

Depression adversely affects health outcomes in patients with childhood-onset systemic lupus erythematous (cSLE). By identifying patients with depressive symptoms, we can intervene early with referrals to mental health resources and improve outcomes. The aim of our quality improvement project was to increase and maintain rates of standardized depression screening for youth with cSLE seen within our pediatric rheumatology clinic.

**Methods:**

Patients with cSLE 12 years of age or older seen for routine follow-up at our pediatric rheumatology clinic from September 16, 2019, through December 30, 2022, were offered the Patient Health Questionnaire-9 modified for adolescents (PHQ-A) to screen for depressive symptoms. A multidisciplinary team developed a key driver diagram to plan potential interventions to improve rates of screening. Plan‒Do‒Study‒Act (PDSA) cycles were used to prepare, implement, and evaluate interventions. Notable interventions focused on accurately identifying eligible patients, facilitating bidirectional communication between staff, and integrating and automating screening within the electronic health record (EHR). Statistical process control (SPC) methods were used for data analysis.

**Results:**

The percentage of eligible patient encounters where depression screening was completed increased from 0 to 81% and was maintained for more than 6 months. This represents special cause variation, as evidenced by data shifts on our statistical process control chart. Among the 592 patients who completed depression screens, 114 (17%) were positive for moderate to severe symptoms, and 59 (9%) were positive for suicidal ideation (SI).

**Conclusions:**

A high rate of standardized depression screening for youth with cSLE was achieved and maintained via integration and automation within our EHR. Establishing a highly reliable screening system is a critical first step in improving mental health care for this vulnerable population of youth.

**Supplementary Information:**

The online version contains supplementary material available at 10.1186/s12969-024-01038-3.

## Background

Childhood-onset systemic lupus erythematosus (cSLE) is a chronic autoimmune disease characterized by rapid accrual of multisystem organ damage. The negative impact of cSLE on emotional health is well recognized. Patients with cSLE have high rates of depressive symptoms [1–7] and 5.4 times the odds of having suicidal ideation compared with healthy controls [4]. Compared with those with adult-onset systemic lupus erythematosus (SLE), patients with cSLE have a greater risk of major depression, independent of both disease activity and duration [6]. Patients with cSLE with psychiatric comorbidities also have lower rates of primary care physician (PCP) visits [4], higher rates of emergency visits [8], higher rates of medication nonadherence [2, 9], and poorer health-related quality of life [10]. Despite this, the majority of patients with cSLE found to have psychiatric diagnoses are not connected with psychiatric services [8]. Therefore, identifying patients with cSLE with symptoms of depression has the potential to improve outcomes through early referral to mental health services.

Both the American Academy of Pediatrics (AAP) and the United States Preventive Services Task Force (USPSTF) recommend annual depression screening for all children 12 years and older [11, 12]. While screening is traditionally performed by primary care providers (PCPs) at health maintenance visits, patients with SLE visit their rheumatologist almost twice as often as their PCPs [4]. Thus, pediatric rheumatologists have a unique opportunity to offer more frequent screening and earlier intervention by implementing depression screening at routine clinic visits. While pediatric rheumatologists agree that mental health screening is important, only 2% of practices routinely screen for mental health disorders [13].

A few prior studies have shown the feasibility and acceptability of screening for depression and suicidal ideation in the pediatric rheumatology clinic [7, 14, 15]. However, none have demonstrated high rates of depression screening for patients with cSLE via the implementation of an automated, electronic health record (EHR) driven screening system. Through rigorous application of quality improvement (QI) methodology, we sought to improve the quality of care provided to youth with cSLE at Monroe Carell Jr. Children’s Hospital at Vanderbilt Pediatric Rheumatology Clinic by implementing and automating a system for depression screening. Our specific aim was to increase and sustain rates of standardized depression screening for patients with cSLE presenting for routine follow-up appointments at our pediatric rheumatology clinic from a baseline of 0% to 80% by December 30th, 2022.

## Methods

### Clinical setting and patient population

This project took place in a hospital-based pediatric rheumatology clinic at a moderate-sized, urban, pediatric tertiary care center in the southeastern United States. Patient care in our clinic is provided by a multidisciplinary team including five attending pediatric rheumatologists, three fellows, three nurse practitioners, three nurse case managers, a physical therapist, an occupational therapist, and a social worker. Outreach clinics were excluded because of a lack of required support staff at these sites (i.e. social workers, emergency rooms, and dedicated rheumatology nurses). We serve a diverse population of patients with cSLE, ranging in age from children to young adults. According to self-reported data within the EHR, most of our patient population is female (86%) and of African American (47%), Caucasian (39%), Asian (9%), or Hispanic (0.8%) race. Our patients with cSLE have follow-up visits as frequently as once per week to once every 6 months, depending on disease activity and severity. In a prior study performed in a cohort of patients with cSLE seen at our center, Systemic Lupus Erythematosus Disease Activity Index (SLEDAI) scores ranged from 2–8 with an average of 5.1 [2]. Our site utilizes Epic (EpicCare Ambulatory, Epic Systems Corporation, Verona, WI, USA) as the EHR.

### Quality improvement methodology

**Fig. 1:**
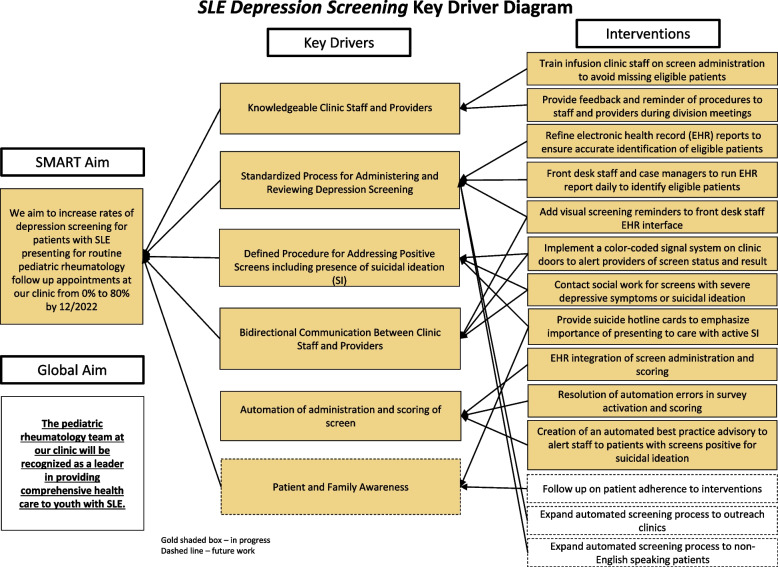
Key driver diagram

We used the Institute for Healthcare Improvement’s (IHI) Model for Improvement [16] to structure and execute our QI project. The IHI Model for Improvement is an accepted framework used to accelerate process improvement within healthcare settings [17]. Through application of this model, we identified key drivers that contribute to achieving a reliable mental health screening system. Our key drivers included knowledgeable clinic staff and providers, a standardized process for administering screens and reviewing screen results, a defined process for addressing positive screens and suicidal ideation, bidirectional communication between clinic staff and providers, EHR automation of the screening process, and patient and family awareness (Fig. 1). We then developed interventions that directly related to these key drivers. Per the Model for Improvement, these interventions were iteratively planned, tested, and adapted using Plan-Do-Study-Act (PDSA) cycles to ensure changes to the process resulted in improvement of our desired metric – the rate of standardized depression screening for patients with cSLE presenting for routine follow-up appointments at our pediatric rheumatology clinic.

Our QI team included an attending pediatric rheumatologist, a clinic nurse, nurse case managers, a pediatric resident, a pediatric rheumatology fellow, an Epic physician builder, a social worker, a member of our front desk staff, and a discharge coordinator. The QI team met biweekly to review the results of each intervention via run charts, control charts, and qualitative feedback from members of the QI team. The team used concepts of highly reliable systems [17] to develop and refine workflow processes for each PDSA cycle. Qualitative feedback was also elicited from providers and patients to gauge satisfaction and plan future process improvements. The Standards for Quality Improvement Reporting Excellence (SQUIRE 2.0) guidelines were used in the preparation of this manuscript [18]. The Vanderbilt University Medical Center institutional review board (IRB) reviewed the proposed project and deemed it exempt from IRB oversight as a QI activity (IRB #190,126).

### Screening, scoring, and treatment algorithm

We used the Patient Health Questionnaire-9 modified for adolescents (PHQ-A) to screen for depressive symptoms. This is a standardized screening tool for depressive symptoms validated for ages 12–18 years. In validation studies, the PHQ-A was found to be self-administered in less than 5 min [19]. The sensitivity and specificity of the PHQ-A for major depressive disorder is 75% and 94% respectively. We chose this screening tool due to its specific age validation for adolescents as compared to the PHQ-9, its fast self-administration, and its wide use in clinical and research settings in pediatric rheumatology [2, 4, 7, 19–23]. The screen was offered to patients identified with cSLE using specified International Classification of Diseases 10th Revision (ICD-10) codes (Supplemental Table 1), who presented to our clinic for a follow-up visit. Patients who were greater than or equal to 12 years of age and English-speaking were eligible, in accordance with the validation parameters of the PHQ-A and the limited availability of translated screening materials at our institution upon initiation of the project.

The PHQ-A scoring algorithm and suggested treatment plan are illustrated in Supplemental Fig. 1. For patients with moderate depressive symptoms or higher, a referral to a mental health provider was advised. Patients with severe depressive symptoms were seen by a social worker for suicide risk assessment and safety planning. Patients who were deemed actively suicidal were referred to the emergency department for further evaluation and urgent intervention. We performed frequent reviews of our data to ensure that these appropriate interventions were taking place in response to positive screens.

### Measures and analysis

The primary outcome measure was the percentage of eligible patient encounters for which depression screening was offered using the PHQ-A screening tool. Eligible patient encounters were identified by an EHR report of patient encounters within the pediatric rheumatology clinic during two-week intervals with a documented clinic visit billing code related to SLE as defined by the ICD-10 codes previously described. The screening results were manually extracted and reviewed by members of the QI team (V.N., A.D., E.D., N.B., S.P.) to determine if the PHQ-A was completed at each visit and if appropriate clinical actions were taken.

The primary outcome measure was plotted on an SPC chart (P-chart) using a mean centerline and upper and lower control limits to assess for special cause variation. Special cause variation is reflective of specific circumstances that led to improvements in our system compared with common cause variation, which reflects causes of variation inherent in the system itself [24]. Statistical rules are used to identify nonrandom patterns on a control chart that determine special cause variation [24]. These rules include (1) a single point outside the control limits, (2) a run of eight or more points in a row above (or below) the centerline (shift), (3) six consecutive points increasing (trend up) or decreasing (trend down), (4) two out of three consecutive points near (outer one third) a control limit, and (5) fifteen consecutive points close (inner one third of the chart) to the centerline [24].

We also collected and analyzed secondary data using descriptive statistical methods to help inform ongoing efforts aimed at improving mental health care for this population. We measured the total number of completed PHQ-A screens, the percentage of positive PHQ-A screens, defined as those indicating moderate to severe depressive symptoms, and the percentage of screens with positive suicidal ideation for which clinically appropriate actions were taken. We also measured the number of individual patients with suicidal ideation.

### Interventions

**Fig. 2:**
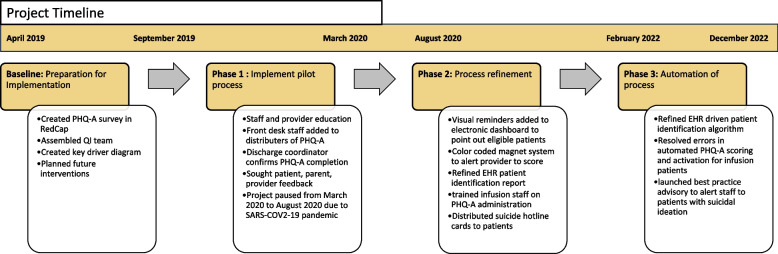
Timeline

Prior to the initiation of this project, we were not administering mental health screening questionnaires in our clinic. Our project was completed in three phases over a four-year period as illustrated in Fig. 2.

### Phase 1

In this phase, we focused on creating a standardized process to administer the PHQ-A with defined safety protocols to address positive screens. Nurse case managers used an EHR generated report to perform pre-visit planning to identify eligible patient visits on a weekly basis. These nurses and front desk staff provided the PHQ-A to patients on an electronic tablet at the time of in-person check-in for their appointment. Patients completed the screen prior to starting the provider visit. Thus, no additional time was taken during the visit for completion of the screen. The PHQ-A form was provided via Research Electronic Data Capture (REDCap), a secure-HIPPA-compliant web-based software platform [25, 26]. We chose to provide our screening tool via an electronic tablet rather than on paper to maintain patient confidentiality and facilitate immediate manual electronic upload of the result to the EHR for provider review. Nurse case managers, who collected the tablets upon completion of the screen and uploaded the results to the EHR, also reviewed the screen results and helped verbally notify the provider of the score. During the visit, the visit provider and/or social worker were expected to address a positive screen. The project was paused at the start of the SARS-CoV-2 pandemic due to safety concerns amid an increase in telehealth visits and a change in clinic staffing protocols.

### Phase 2

The second phase of our project focused on refining our screening process to increase the reliability of our system. We focused on interventions that would reduce the dependence of our process on specific individuals, improve bidirectional communication among clinic staff, and bolster safety mechanisms.

### Phase 3

The third phase of our project focused on the automation of PHQ-A screening and safety procedures through the EHR to further improve reliability and promote sustainability by decreasing the dependence of our process on clinical staff. The EHR was programmed to automatically assign and display the PHQ-A as a pre-visit screening questionnaire at eligible patient visits. The screen was still completed by the patient on a tablet after in-person check-in, before starting the provider visit. The EHR then scored the screen and displayed the results in an easily visible location within the visit encounter. Similar to the prior phases, the visit provider and/or social worker were expected to address a positive screen during the visit. The PHQ-A was only visible to patients at the time of their in-person check-in rather than via the patient portal a few days before their appointment. This was intentionally done to ensure that the results were immediately reviewed upon completion of the screen to protect the safety of our patients. Another safety mechanism was the best practice advisory alert (BPA), which was created to alert staff to patients with suicidal ideation upon opening the chart. A rheumatology physician in our group who is an Epic physician builder (L.B.) helped build this system.

## Results

### Primary measure: rates of completed depression screening at eligible visits

**Fig. 3:**
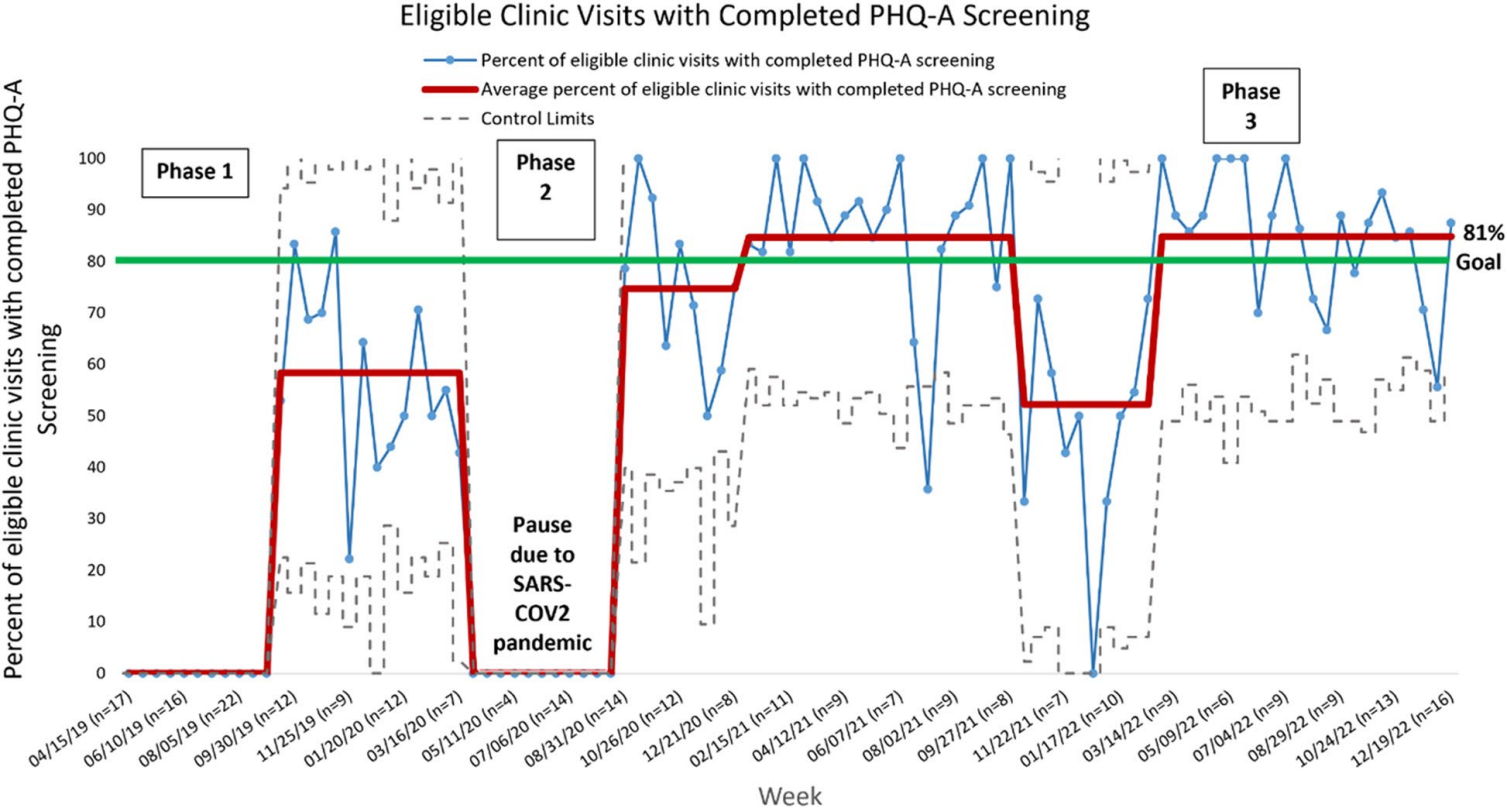
Control chart (P Chart)

In phase 1, the percentage of eligible patient encounters where depression screening was completed increased from 0 to 58%. This was illustrated by a data shift representing special cause variation. However, there was significant variation in our process, as evidenced by a wide gap between the upper and lower control limits on our SPC chart (Fig. 3). Key interventions during this phase included pre-visit planning to identify patients, education of clinic staff, and involving front desk staff and discharge coordinators in administering tablets and ensuring screen completion, respectively.

In phase 2, in response to process refinements, two data shifts occurred on our control chart in the direction of our goal. The percentage of eligible patient encounters where depression screening was completed increased from 58 to 85%, above our goal of 80%. Key interventions included displaying visual cues within the EHR and on clinic doors to remind providers of screening needs and quickly communicate screen severity, refining the pre-visit planning system, educating our infusion center staff, and handing out suicide hotline cards to patients. There was a subsequent data shift that occurred in the direction opposite our goal, with rates of depression screening dropping to 52%. This took place during a maintenance period of our project where we were not actively implementing new interventions. In addition, one of our nurse case managers, who helped identify patients and administer and review screens, transitioned to a different role outside of our department.

In phase 3, almost immediately after automating our PHQ-A screening process via the EHR, our rates of completed depression screening increased from 52 to 81%, as evidenced by another data shift on our control chart. This rate was maintained for 9 months until the end of our observation period. Successful interventions during this phase included refinement of the EHR patient identification algorithm, resolution of coding errors, and creation of a BPA alert in the chart which alerted staff to patients with suicidal ideation.

### Secondary data: prevalence of positive depression screens and rates of appropriate intervention

There was an average of 12 eligible encounters for screening per 2-week period. During the project period, there were 886 eligible encounters for patients with cSLE aged 12 years or older presenting for a follow-up visit. There were 6 patients seen in our clinic during the project period who had a preferred language other than English. Most of these patients had a preferred language of Spanish. Depression screening was assigned at 661 (75%) of the eligible encounters. Among these assigned screens, 592 were completed and 69 were declined by the patient. Among the 592 completed depression screens, 114 (17%) were positive, as defined by a score indicating moderate depressive symptoms or higher. There were 349 (53%) with no symptoms, 129 (20%) with mild symptoms, 40 (6%) with moderate symptoms, 10 (2%) with high symptoms, 64 (10%) with severe symptoms and 59 (9%) with suicidal ideation.

Suicidal ideation was identified in 21 individual patients. In phases 1 and 2, appropriate actions were taken for 100% of patients with suicidal ideation. These actions included social work involvement, home safety planning, referral to psychiatry, and referral to the emergency department if indicated. In phase 3, appropriate actions were taken for 92% (11/12) of patients with suicidal ideation. One patient with suicidal ideation did not receive a specific intervention for SI on the day of the clinic visit. This was likely due to their screener not being automatically scored. This took place within the first few weeks of initiation of our automated process. This patient had met with our social worker at a clinic visit one week prior for SI. The patient denied active SI at that time and had been provided with mental health resources to establish local care. This programing error was quickly resolved, and shortly thereafter, our best practice advisory was introduced to the EHR to automatically identify and flag patients with suicidal ideation upon opening of the chart. During the remainder of the study period, all patients with suicidal ideation received appropriate intervention.

### Qualitative feedback

Qualitative feedback was elicited from providers and patients regarding our screening process. Providers in our clinic reported satisfaction with the process. They identified depressive symptoms in patients with whom they otherwise would not have addressed depression, stating that “patient[s] that I did not suspect any mood disturbance and would not have asked about mood or depression screened positive—this further enforces why we should screen all patients with chronic disease for depression.” Another provider noted the importance of making the screening process automatic: “Our patients with SLE are complex with many moving parts, numerous medications, and multiple involved organs—it is easy to forget to evaluate mental health.” Patients also responded favorably to the screening, saying “I am glad that we are testing this. It shows growth." One guardian said, “I am happy that you all are screening for overall health.” We did not receive negative feedback from patients and families regarding use of the tablet for survey completion. We did have patients who declined to complete the screen or did not understand the screen. There was no formal exclusion of these patients in light of this being a QI project occurring in the context of routine clinical care.

## Discussion

Early identification and treatment of depression has the potential to improve outcomes for youth with cSLE. Pediatric rheumatologists are uniquely positioned to provide depression screening and facilitate mental health treatment plans for youth with cSLE because of the frequency of patient encounters and ongoing therapeutic relationships with patients and families. Symptoms of depression are often subtle and can be missed despite a patient’s multiple interactions with the health care system. Therefore, pediatric rheumatologists need to develop strategies for implementing standardized mental health screening into routine clinical care, as was emphasized in the Childhood Arthritis and Rheumatology Research Alliance (CARRA) Mental Health Workgroup prioritized agenda to improve mental health in pediatric rheumatology patients [27]. Prior studies have shown the feasibility and acceptability of screening for depression and suicidal ideation in pediatric rheumatology clinics [7, 14, 15]. To our knowledge, this is the first study to implement standardized depression screening via a fully integrated and automated EHR system for youth with cSLE seen within a pediatric rheumatology clinic as a component of routine clinical care.

Through rigorous application of QI methodology, we were able to increase rates of standardized depression screening for youth with cSLE in our pediatric rheumatology clinic from 0 to 81%. In addition, key change concepts that contributed to achieving and maintaining high rates of depression screening were identified. In phase 1, we created a feasible process. However, rates of completed depression screening remained below our goal, with significant variation in our process due to high dependence on individual clinic staff. In phase 2, we exceeded our goal screening rate through a series of process refinements that supported bi-directional communication between clinic staff members, broadened the involvement of staff members, and addressed missed screening opportunities for patients receiving coordinated services. However, the decrease in screening rates we encountered during the maintenance period of phase 2 demonstrated a lack of process sustainability due to a persistently person-dependent system. In phase 3, we quickly achieved and maintained rates of depression screening above our goal of 80% by integrating and automating our screening process via the EHR, thereby reducing dependency on clinic staff. This high rate of screening was maintained with minimal staff effort over a 9-month period. The programming errors encountered in this phase exposed the limitations of automation. They demonstrated the importance of monitoring our results frequently during process changes. The automatic EHR alert for suicidal ideation was essential in ensuring that patients with suicidal ideation would be accurately identified and provided immediate intervention.

In addition to identifying change concepts to support the successful implementation of depression screening into routine pediatric rheumatology care for youth with cSLE, our work adds to the epidemiologic literature describing the degree of depressive symptoms in this population. Almost half of the screen results during our implementation period demonstrated depressive symptoms (mild-severe), with close to 20% showing moderate to severe symptoms. Strikingly, 21 individual patients were identified as having suicidal ideation. These findings are similar to those reported in previous studies where prevalence estimates for depression in cSLE patients range between 20 and 54% [1, 2, 4–6, 28–30] with a disproportionate percentage affected by suicidal ideation compared to healthy controls [2, 4, 29]. The high prevalence of these symptoms emphasizes the importance of using routine screening to identify patients with depressive symptoms, especially those with suicidal ideation, in order to facilitate the development of mental health treatment plans that otherwise may not be created.

Our ability to robustly maintain screening at every routine follow-up visit for patients with cSLE allowed patients and providers to maintain familiarity with the tool and comfort in discussing and interpreting the results. We believe this has helped normalize, and even de-stigmatize, the assessment of mental health needs in our clinic. We also recognize that individual patients have variability in their depressive symptoms over time. By screening at each encounter, we were able to identify depressive symptoms in patients who may not have had concerning screens at previous visits. These observations were further supported by the qualitative feedback we elicited from providers and patients and their families.

The generalizability of our depression screening process may be limited by certain features of our system. First, our system requires specific functionalities of the EHR including customized patient identification algorithms, integrated electronic pre-visit surveys, point-of-care visualization of results within the EHR, and the activation of best practice advisory alerts. The feasibility for other centers to implement our process may be limited by the availability of staff with expertise in building new EHR processes and lack of resources such as tablets for patients to complete EHR integrated screenings. Second, our system requires robust nursing, front desk, and discharge coordinator support to ensure adequate completion of the screening process, and in-person social work support to ensure positive screens are addressed safely. Other pediatric rheumatology centers may not have this level of ancillary support staff. In fact, a recent survey revealed that fewer than half of the pediatric rheumatology clinics in North America had access to a social worker [31]. Nonetheless, the change concepts that proved successful during each phase of our project are adaptable to any system and can help guide similar work at other institutions.

There are limitations of our current depression screening process that need to be addressed. We recognize that excluding satellite clinics, given the lack of on-site social work support to protect patient safety at these locations, adversely affects the equity of our system by disproportionately affecting rural and underserved patients in our state. While it is not feasible to dispatch already limited social workers to satellite clinics, the utilization of telehealth systems (recently optimized during the SARS-CoV-2 pandemic) may offer a solution to expand access to social work services at these sites. Similarly, we were not able to screen patients with limited English proficiency because of a lack of translated materials. Now that our institution has electronic PHQ-A screens in Spanish, we plan to implement screening for this patient population. We will continue to advocate for translation to additional languages as specific needs are identified. Lastly, we recognize that time added to address screen results within the clinic visit is an important variable to consider when implementing mental health screening. Unfortunately, we were not able to collect accurate data on this balancing measure. There is EHR capability to calculate time spent in the visit based on status changes (i.e. ready for provider, provider in room), but status changes must be manually selected by providers and so are not consistently used within our current clinic workflow.

While our system was created for a single patient population within our single-center pediatric rheumatology clinic, its use could be expanded to clinics that care for other patient populations with chronic diseases for whom addressing mental health concerns is a priority [32–34]. Within our clinic, we plan to expand routine depression screening to patients with juvenile dermatomyositis (JDM), a population that has also been shown to report high levels of emotional distress as well as self-reported and clinician-diagnosed anxiety and depression [35–37]. We also plan to implement routine anxiety screening for patients with cSLE and JDM through a similar EHR automated process. Furthermore, we are working to develop a process to ensure that patients with positive screens access the recommended mental health resources to which they are referred. Other centers have also shown that imbedding psychology services within the rheumatology clinic can lead to increased access to mental health treatment [15]. Our clinic is currently in the process of adding a psychology provider to our rheumatology care team.

## Conclusions

We demonstrated the successful implementation of a standardized and sustainable EHR integrated process for depression screening for patients with cSLE using QI methodology. Our work emphasizes the prevalence of depressive symptoms in patients with cSLE. Future work should focus on expansion of mental health screening and integration of services into pediatric rheumatology clinics to improve access to and coordination of mental health care. Furthermore, we advocate for future research studies that will expand beyond the scope of this QI project to: 1) delineate relationships between mental health and disease characteristics for youth with cSLE; 2) quantify the impact of mental health screening and resulting intervention on disease outcomes for youth with cSLE.

## Supplementary Information

Supplementary Material 1: Figure 1. Flow chart of suggested treatment plan based on PHQ-A scores.

Supplementary Material 2: Table 1. International Classification of Diseases 10^th^ Revision codes for categories of systemic lupus erythematous disorders.

## Data Availability

The datasets used and/or analyzed during the current study are available from the corresponding author on reasonable request.

## Abbreviations

cSLE: Childhood-onset systemic lupus erythematosus
PHQ-A: Patient Health Questionnaire-9 modified for adolescents
PDSA: Plan‒Do‒Study‒Act
EHR: Electronic health record
SPC: Statistical process control
SI: Suicidal ideation
SLE: Systemic lupus erythematosus
PCP: Primary care physician
AAP: American Academy of Pediatrics
USPSTF: United States Preventive Services Task Force
QI: Quality improvement
SQUIRE 2.0: Standards for Quality Improvement Reporting Excellence
IRB: Institutional review board
ICD-10: International Classification of Diseases 10^th^ Revision codes
REDCap: Research Electronic Data Capture
BPA: Best practice advisory
CARRA: Childhood Arthritis and Rheumatology Research Alliance
JDM: Juvenile dermatomyositis
