# Supplementary figures and images for: Increasing and maintaining rates of standardized depression screening in youth with childhood-onset systemic lupus erythematosus in a pediatric rheumatology clinic
Source: Pediatr Rheumatol Online J. 2025 Jan 4;23:3. doi: 10.1186/s12969-024-01038-3 (PMC11699778; doi:10.1186/s12969-024-01038-3)

## Slide 1
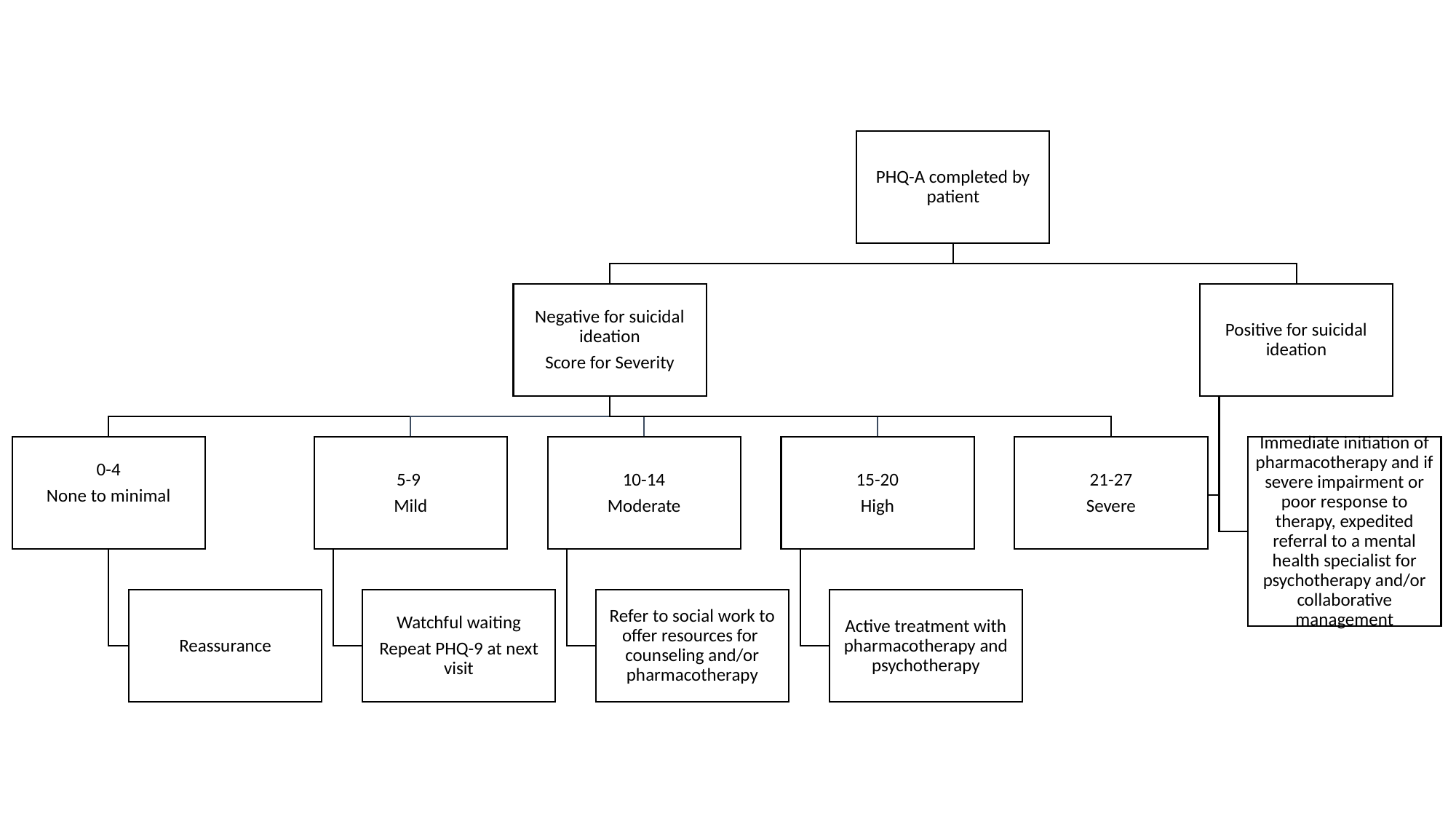

Supplement: Supplementary file 1 — Supplementary Material 1: Figure 1. Flow chart of suggested treatment plan based on PHQ-A scores. [file 12969_2024_1038_MOESM1_ESM.pptx]
