# Supplementary material for: Increasing and maintaining rates of standardized depression screening in youth with childhood-onset systemic lupus erythematosus in a pediatric rheumatology clinic
Source: Pediatr Rheumatol Online J. 2025 Jan 4;23:3. doi: 10.1186/s12969-024-01038-3 (PMC11699778; doi:10.1186/s12969-024-01038-3)
Supplement: Supplementary file 2 — Supplementary Material 2: Table 1. International Classification of Diseases 10th Revision codes for categories of systemic lupus erythematous disorders. [file 12969_2024_1038_MOESM2_ESM.docx]

Supplemental Table 1: International Classification of Diseases 10^th^ Revision codes for categories of systemic lupus erythematous disorders

| **SLE Disease Category** | **ICD-10-CM** |
| --- | --- |
| Systemic lupus erythematosus (CMS/HCC) | M32.9 |
| Drug-induced systemic lupus erythematosus | M32.0 |
| Lung involvement in systemic lupus erythematosus | M32.13 |
| Systemic lupus erythematosus, unspecified | M32.9 |
| Glomerular disease in systemic lupus erythematosus | M32.14 |
| Systemic lupus erythematosus, organ or system involvement unspecified | M32.10 |
| Tubulo-interstitial nephropathy in systemic lupus erythematosus | M32.15 |
| Other organ or system involvement in systemic lupus erythematosus | M32.19 |
| Other forms of systemic lupus erythematosus | M32.8 |
| Disseminated lupus erythematosus (CMS/HCC) | M32.9 |
| Lupus disease of the lung | M32.13 |
| Nonbacterial verrucal endocardiosis (CMS/HCC) | M32.11 |
| Atypical verrucal endocarditis (CMS/HCC) | M32.11 |
| Lupus hepatitis (CMS/HCC) | K75.4 |
| SLE glomerulonephritis syndrome (CMS/HCC) | M32.14 |
| SLE glomerulonephritis syndrome, WHO class I (CMS/HCC) | M32.14 |
| SLE with normal kidneys (CMS/HCC) | M32.9 |
| SLE glomerulonephritis syndrome, WHO class II (CMS/HCC) | M32.14 |
| SLE with mesangial proliferative glomerulonephritis (CMS/HCC) | M32.14 |
| SLE glomerulonephritis syndrome, WHO class III (CMS/HCC) | M32.14 |
| SLE with focal and segmental proliferative glomerulonephritis (CMS/HCC) | M32.14 |
| SLE glomerulonephritis syndrome, WHO class IV (CMS/HCC) | M32.14 |
| SLE with diffuse proliferative glomerulonephritis (CMS/HCC) | M32.14 |
| SLE glomerulonephritis syndrome, WHO class V (CMS/HCC) | M32.14 |
| Membranous lupus glomerulonephritis (CMS/HCC) | M32.14 |
| SLE with membranous glomerulonephritis (CMS/HCC) | M32.14 |
| SLE glomerulonephritis syndrome, WHO class VI (CMS/HCC) | M32.14 |
| Lupus with glomerular sclerosis (CMS/HCC) | M32.14 |
| SLE with advanced sclerosing glomerulonephritis (CMS/HCC) | M32.14 |
| Lupus erythematosus disseminatus (CMS/HCC) | M32.9 |
| Lupus glomerulonephritis (CMS/HCC) | M32.14 |
| Lupus nephritis (CMS/HCC) | M32.14 |
| Endocarditis, atypical, verrucous (CMS/HCC) | M32.11 |
| Endocarditis, nonbacterial, verrucous (CMS/HCC) | M32.11 |
| Hepatitis, lupoid (CMS/HCC) | K75.4 |
| Libman Sacks endocarditis (CMS/HCC) | M32.11 |
| Libman-Sacks disease (CMS/HCC) | M32.11 |
| Libman-Sacks disease or syndrome (CMS/HCC) | M32.11 |
| DLE (disseminated lupus erythematosus) (CMS/HCC) | M32.9 |
| SLE (systemic lupus erythematosus) (CMS/HCC) | M32.9 |
| Systemic lupus erythematosus (SLE) | M32 |
| Systemic lupus erythematosus with organ or system involvement | M32.1 |
| Atypical verrucous endocarditis (CMS/HCC) | M32.11 |
| Diffuse lupus glomerulonephritis syndrome (CMS/HCC) | M32.14 |
| Libman-Sacks endocarditis (CMS/HCC) | M32.11 |
| Lupus hepatitis syndrome (CMS/HCC) | K75.4 |
| Lupus panniculitis | L93.2 |
| Membranous lupus nephritis syndrome (CMS/HCC) | M32.14 |
| Nonbacterial verrucous endocarditis (CMS/HCC) | M32.11 |
| Lupus (systemic lupus erythematosus) (CMS/HCC) | M32.9 |
| Lupoid hepatitis (CMS/HCC) | K75.4 |
| Lupus glomerular disease (CMS/HCC) | M32.14 |
| Lupus encephalitis (CMS/HCC) | M32.19 |
| Lupus with encephalitis (CMS/HCC) | M32.19 |
| Systemic lupus erythematosus with encephalitis (CMS/HCC) | M32.19 |
| ESRD (end-stage renal disease) due to SLE (CMS/HCC) | M32.14, N18.6 |
| Systemic lupus erythematosus encephalitis (CMS/HCC) | M32.19 |
| Drug-induced systemic lupus erythematosus (CMS/HCC) | M32.0 |
| Systemic lupus erythematosus complicating pregnancy (CMS/HCC) | O99.89, M32.9 |
| Renal disease associated with lupus (CMS/HCC) | M32.8 |
| Kidney disease associated with lupus (CMS/HCC) | M32.8 |
| Systemic lupus (CMS/HCC) | M32.9 |
| Maternal systemic lupus erythematosus, antepartum (CMS/HCC) | O99.89, M32.9 |
| Systemic lupus erythematosus, maternal, antepartum (CMS/HCC) | O99.89, M32.9 |
| Pulmonary disease in systemic lupus erythematosus (CMS/HCC) | M32.13 |
| Lung disease with systemic lupus erythematosus (CMS/HCC) | M32.13 |
| Pulmonary disease in systemic lupus (CMS/HCC) | M32.13 |
| Lupus cerebritis (CMS/HCC) | M32.19 |
| Systemic lupus erythematosus arthritis (CMS/HCC) | M32.9 |
| Lupus arthritis (CMS/HCC) | M32.9 |
| Nephritis in lupus (CMS/HCC) | M32.14 |
| Stage I lupus nephritis (WHO) (CMS/HCC) | M32.14 |
| Stage II lupus nephritis (WHO) (CMS/HCC) | M32.14 |
| Stage III lupus nephritis (WHO) (CMS/HCC) | M32.14 |
| Stage IV lupus nephritis (WHO) (CMS/HCC) | M32.14 |
| Stage V lupus nephritis (WHO) (CMS/HCC) | M32.14 |
| Lung involvement in systemic lupus erythematosus (CMS/HCC) | M32.13 |
| Glomerular disease in systemic lupus erythematosus (CMS/HCC) | M32.14 |
| Tubulo-interstitial nephropathy in systemic lupus erythematosus (CMS/HCC) | M32.15 |
| End stage lupus glomerulonephritis (CMS/HCC) | M32.14 |
| Exacerbation of systemic lupus erythematosus (CMS/HCC) | M32.9 |
| Exacerbation of systemic lupus (CMS/HCC) | M32.9 |
| SLE exacerbation (CMS/HCC) | M32.9 |
| Central nervous system lupus (CMS/HCC) | M32.19 |
| CNS lupus (CMS/HCC) | M32.19 |
| Lupoid nephritis (CMS/HCC) | M32.10, N08 |
| Systemic lupus complicating pregnancy (CMS/HCC) | O99.89, M32.9 |
| Chronic lupus nephritis (CMS/HCC) | M32.14 |
| Diffuse proliferative lupus glomerulonephritis (CMS/HCC) | M32.14 |
| Systemic lupus erythematosus with organ system involvement (CMS/HCC) | M32.10 |
| Systemic lupus with central nervous system involvement (CMS/HCC) | M32.19 |
| Neuropathy due to systemic lupus erythematosus (CMS/HCC) | M32.9, G63 |
| Neuropathy due to SLE (systemic lupus erythematosus) (CMS/HCC) | M32.9, G63 |
| End-stage renal disease due to systemic lupus erythematosus (CMS/HCC) | M32.14, N18.6 |
| Systemic lupus erythematosus with focal and segmental proliferative glomerulonephritis (CMS/HCC) | M32.14 |
| WHO class VI systemic lupus erythematosus glomerulonephritis syndrome (CMS/HCC) | M32.14 |
| WHO class IV systemic lupus erythematosus glomerulonephritis syndrome (CMS/HCC) | M32.14 |
| WHO class V systemic lupus erythematosus glomerulonephritis syndrome (CMS/HCC) | M32.14 |
| WHO class I systemic lupus erythematosus glomerulonephritis syndrome (CMS/HCC) | M32.14 |
| WHO class II systemic lupus erythematosus glomerulonephritis syndrome (CMS/HCC) | M32.14 |
| Systemic lupus erythematosus complicating pregnancy in second trimester (CMS/HCC) | O99.89, M32.9 |
| Systemic lupus erythematosus complicating pregnancy in third trimester (CMS/HCC) | O99.89, M32.9 |
| Systemic lupus erythematosus complicating pregnancy in first trimester (CMS/HCC) | O99.89, M32.9 |
| Acute systemic lupus erythematosus (CMS/HCC) | M32.9 |
| Systemic lupus erythematosus (SLE) in pediatric patient (CMS/HCC) | M32.9 |
| Systemic lupus erythematosus (SLE) in adult (CMS/HCC) | M32.9 |
| Systemic lupus erythematosus (SLE) affecting pregnancy, antepartum (CMS/HCC) | O99.89, M32.9 |
| Systemic lupus erythematosus affecting pregnancy in first trimester (CMS/HCC) | O99.89, M32.9 |
| Systemic lupus erythematosus affecting pregnancy (CMS/HCC) | O99.89, M32.9 |
| Systemic lupus erythematosus affecting pregnancy in third trimester (CMS/HCC) | O99.89, M32.9 |
| Systemic lupus erythematosus affecting pregnancy in second trimester (CMS/HCC) | O99.89, M32.9 |
| Pericarditis associated with systemic lupus erythematosus (CMS/HCC) | I31.9, M32.12 |
| Nephropathy due to systemic lupus erythematosus (SLE) (CMS/HCC) | M32.14 |
| ISN/RPS class I glomerulonephritis due to systemic lupus erythematosus (SLE) (CMS/HCC) | M32.14 |
| Lupus nephritis, ISN/RPS class I (CMS/HCC) | M32.14 |
| ISN/RPS class II glomerulonephritis due to systemic lupus erythematosus (SLE) (CMS/HCC) | M32.14 |
| ISN/RPS class III glomerulonephritis due to systemic lupus erythematosus (SLE) (CMS/HCC) | M32.14 |
| Lupus nephritis, ISN/RPS class III (CMS/HCC) | M32.14 |
| Lupus nephritis, ISN/RPS class IV (CMS/HCC) | M32.14 |
| Lupus nephritis, ISN/RPS class V (CMS/HCC) | M32.14 |
| ISN/RPS class VI glomerulonephritis due to systemic lupus erythematosus (SLE) (CMS/HCC) | M32.14 |
| Lupus nephritis, ISN/RPS class II (CMS/HCC) | M32.14 |
| ISN/RPS class IV glomerulonephritis due to systemic lupus erythematosus (SLE) (CMS/HCC) | M32.14 |
| ISN/RPS class V glomerulonephritis due to systemic lupus erythematosus (SLE) (CMS/HCC) | M32.14 |
| Lupus nephritis, ISN/RPS class VI (CMS/HCC) | M32.14 |
| Systemic lupus erythematosus (SLE) with pericarditis (CMS/HCC) | M32.12 |
| Systemic lupus erythematosus of mother during pregnancy (CMS/HCC) | O26.899, M32.9 |
| Systemic lupus erythematosus, unspecified (CMS/HCC) | M32.9 |
| Systemic lupus erythematosus, organ or system involvement unspecified (CMS/HCC) | M32.10 |
| Other organ or system involvement in systemic lupus erythematosus (CMS/HCC) | M32.19 |
| Other forms of systemic lupus erythematosus (CMS/HCC) | M32.8 |
| Systemic lupus erythematosus complicating pregnancy, second trimester | O26.892, M32.9 |
| Systemic lupus erythematosus complicating pregnancy, third trimester | O26.893, M32.9 |
| Systemic lupus erythematosus complicating pregnancy, first trimester | O26.891, M32.9 |
| Systemic lupus erythematosus complicating pregnancy, unspecified trimester | O26.899, M32.9 |
| Systemic lupus complicating pregnancy, third trimester | O26.893, M32.9 |
| Systemic lupus complicating pregnancy, first trimester | O26.891, M32.9 |
| Systemic lupus complicating pregnancy, second trimester | O26.892, M32.9 |
| Systemic lupus complicating pregnancy, unspecified trimester | O26.899, M32.9 |
| Pericarditis associated with systemic lupus erythematosus, unspecified chronicity (CMS/HCC) | I31.9, M32.12 |
| Other systemic lupus erythematosus with endocarditis (CMS/HCC) | M32.11 |
| Drug-induced systemic lupus erythematosus with other organ involvement (CMS/HCC) | M32.0, M32.10 |
| Other systemic lupus erythematosus with tubulo-interstitial nephropathy (CMS/HCC) | M32.15 |
| Other systemic lupus erythematosus with pericarditis (CMS/HCC) | M32.12 |
| Other systemic lupus erythematosus with lung involvement (CMS/HCC) | M32.13 |
| Other systemic lupus erythematosus with other organ involvement (CMS/HCC) | M32.19 |
| Other systemic lupus erythematosus with glomerular disease (CMS/HCC) | M32.14 |
| Systemic lupus erythematosus with lung involvement, unspecified SLE type (CMS/HCC) | M32.13 |
| Systemic lupus erythematosus (SLE) with pericarditis, unspecified SLE type (CMS/HCC) | M32.12 |
| Drug-induced systemic lupus erythematosus, unspecified organ involvement status (CMS/HCC) | M32.0 |
| Systemic lupus erythematosus, unspecified SLE type, unspecified organ involvement status (CMS/HCC) | M32.9 |
| Systemic lupus erythematosus with glomerular disease, unspecified SLE type (CMS/HCC) | M32.14 |
| Systemic lupus erythematosus with endocarditis, unspecified SLE type (CMS/HCC) | M32.11 |
| Systemic lupus erythematosus with other organ involvement, unspecified SLE type (CMS/HCC) | M32.19 |
| Systemic lupus erythematosus with tubulo-interstitial nephropathy, unspecified SLE type (CMS/HCC) | M32.15 |
| Other forms of systemic lupus erythematosus, unspecified organ involvement status (CMS/HCC) | M32.8 |
| Drug-induced systemic lupus erythematosus with lung involvement (CMS/HCC) | M32.0, M32.13 |
| Systemic lupus erythematosus with tubulo-interstitial nephropathy (CMS/HCC) | M32.15 |
| Drug-induced systemic lupus erythematosus with endocarditis (CMS/HCC) | M32.0, M32.11 |
| Drug-induced systemic lupus erythematosus with pericarditis (CMS/HCC) | M32.0, M32.12 |
| Systemic lupus erythematosus with lung involvement (CMS/HCC) | M32.13 |
| Systemic lupus erythematosus with endocarditis (CMS/HCC) | M32.11 |
| Systemic lupus erythematosus with glomerular disease (CMS/HCC) | M32.14 |
| Drug-induced systemic lupus erythematosus with tubulo-interstitial nephropathy (CMS/HCC) | M32.0, M32.15 |
| Drug-induced systemic lupus erythematosus with glomerular disease (CMS/HCC) | M32.0, M32.14 |
| Systemic lupus erythematosus (SLE) in childhood (CMS/HCC) | M32.9 |
| Systemic lupus erythematosus (SLE) with serositis (CMS/HCC) | M32.19 |
| Systemic lupus erythematosus (SLE) with leukopenia (CMS/HCC) | M32.19, D72.819 |
| Neonatal systemic lupus erythematosus (SLE) (CMS/HCC) | P96.89, M32.9 |
| Recurrent class 3 lupus nephritis after kidney transplantation (CMS/HCC) | T86.19, M32.14 |
| Recurrent class 5 lupus nephritis after kidney transplantation (CMS/HCC) | T86.19, M32.14 |
| Recurrent class 2 lupus nephritis after kidney transplantation (CMS/HCC) | T86.19, M32.14 |
| De novo class 2 lupus nephritis after kidney transplantation (CMS/HCC) | T86.19, M32.14 |
| Recurrent class 4 lupus nephritis after kidney transplantation (CMS/HCC) | T86.19, M32.14 |
| De novo class 4 lupus nephritis after kidney transplantation (CMS/HCC) | T86.19, M32.14 |
| De novo class 5 lupus nephritis after kidney transplantation (CMS/HCC) | T86.19, M32.14 |
| De novo class 3 lupus nephritis after kidney transplantation (CMS/HCC) | T86.19, M32.14 |
| Pericarditis in systemic lupus erythematosus | M32.12 |
| Pericarditis secondary to systemic lupus erythematosus (CMS/HCC) | M32.12 |
| Lupus pericarditis (CMS/HCC) | M32.12 |
| Pericarditis in systemic lupus erythematosus (CMS/HCC) | M32.12 |
| Chronic pericarditis associated with systemic lupus erythematosus (SLE) (CMS/HCC) | M32.12 |
| Chronic adhesive pericarditis associated with systemic lupus erythematosus (SLE) (CMS/HCC) | I31.0, M32.12 |
| Chronic constrictive pericarditis associated with systemic lupus erythematosus (SLE) (CMS/HCC) | M32.12 |
| Chronic pericarditis associated with systemic lupus erythematosus (SLE), unspecified complication status (CMS/HCC) | M32.12 |
| Acute pericarditis associated with systemic lupus erythematosus (SLE) (CMS/HCC) | M32.12 |
| Lupus disease of lung (CMS/HCC) | M32.13 |
| Systemic lupus erythematosus (SLE) not affecting current episode of care (CMS/HCC) | M32.9 |
| Bullous systemic lupus erythematosus (SLE) (CMS/HCC) | M32.8 |
| Chorea concurrent with and due to systemic lupus erythematosus (CMS/HCC) | G25.5, M32.19 |
| Demyelination of central nervous system due to systemic lupus erythematosus (SLE) (CMS/HCC) | G37.9, M32.19 |
| Pulmonary disease due to lupus (CMS/HCC) | M32.13 |
| Pulmonary disease due to systemic lupus erythematosus (SLE) (CMS/HCC) | M32.13 |
| Word Health Organization class II systemic lupus erythematosus (SLE) glomerulonephritis syndrome (CMS/HCC) | M32.14 |
| Fulminating systemic lupus erythematosus (SLE) (CMS/HCC) | M32.8 |
| Systemic lupus erythematosus (SLE) with normal kidneys (CMS/HCC) | M32.9 |
| Limited lupus erythematosus | L93.0 |
| Rash due to systemic lupus erythematosus (SLE) (CMS/HCC) | M32.19, R21 |
| Systemic lupus erythematosus (SLE) with multisystem involvement (CMS/HCC) | M32.19 |
